# Coude flottant associant une fracture de Monteggia Bado 4 à une fracture de la palette humérale

**DOI:** 10.11604/pamj.2015.20.257.6529

**Published:** 2015-03-17

**Authors:** Soufiane Guelzim, Mustapha Mahfoud

**Affiliations:** 1 Service de Chirurgie Orthopédique et Traumatologie, CHU Ibn Sina Rabat, Maroc

**Keywords:** Coude flottant, ostéosynthèse, rééducation, floating elbow, osteosynthesis, physiotherapy

## Image en medicine

Le coude flottant représente une association lésionnelle peu fréquente en traumatologie et qui pose des problèmes pronostique et fonctionnel majeurs. D'où l'importance d'un diagnostic précis avec une prise en charge rapide et adéquate des blessés. Une ostéosynthèse stable de l'ensemble des lésions doit être recommandée permettant une rééducation précoce. La hiérarchisation des gestes s'impose en fonction du type anatomopathologique des lésions. Il s'agit d'images cliniques et radiologiques per, pré et post opératoire d'un coude flottant chez un patient de 40 ans suite à un accident de voiture associant une fracture de la palette humérale métaphyso-épiphysaire à une fracture de monteggia type 4 selon la classification de Bado (fractures des deux os de l'avant bras avec luxation de la tête radiale). Traitement chirurgical: voie d'abord postérieure pour le 1er temps: réduction et vissage de la palette humérale profitant de la fracture de l'olécrâne pour une très bonne exposition de la palette humérale. 2ème temps: réduction et ostéosynthèse de la fracture bifocale comminutive du cubitus par 2 plaques vissées dont une en crochet pour l'olécrâne, réduction spontanée de la tête radiale. 3ème temps: réduction et ostéosynthèse de la fracture du radius par une plaque vissée, voie d'abord antéro-externe.

**Figure 1:**
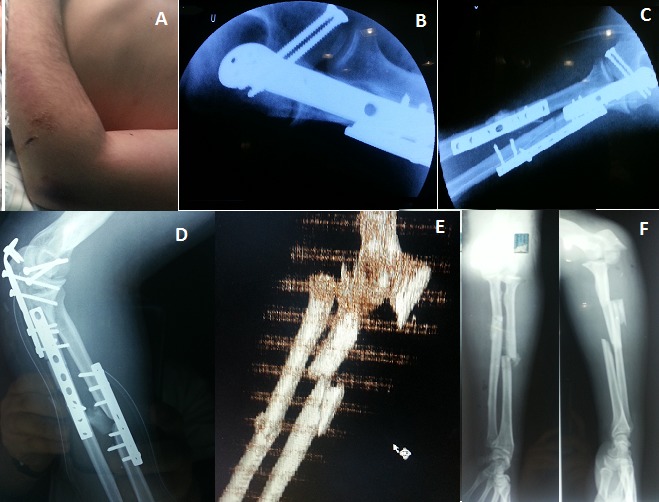
A) image clinique du coude flottant; B) image de scopie per opératoire; C) image de scopie per opératoire; D) radiographie de Contrôle post opératoire; E) TDM avec reconstruction avant bras–coude; F) radiographies standards de l'avant-bras prenant le coude face-profil

